# Supplementary material for: smiFISH and embryo segmentation for single-cell multi-gene RNA quantification in arthropods
Source: Commun Biol. 2021 Mar 19;4:352. doi: 10.1038/s42003-021-01803-0 (PMC7979837; doi:10.1038/s42003-021-01803-0)
Supplement: Supplementary file 2 — Supplementary Information [file 42003_2021_1803_MOESM2_ESM.pdf]

Supplementary Information

smiFISH and embryo segmentation for single-cell multi-gene RNA quantification in arthropods

Llilians Calvo[1]†, Matthew Ronshaugen[1] and Tom Pettini[1]†*

[1]Faculty of Biology, Medicine and Health, The University of Manchester, Manchester, M13 9PT, UK

† These authors contributed equally

* Correspondence: pettini.tom@gmail.com

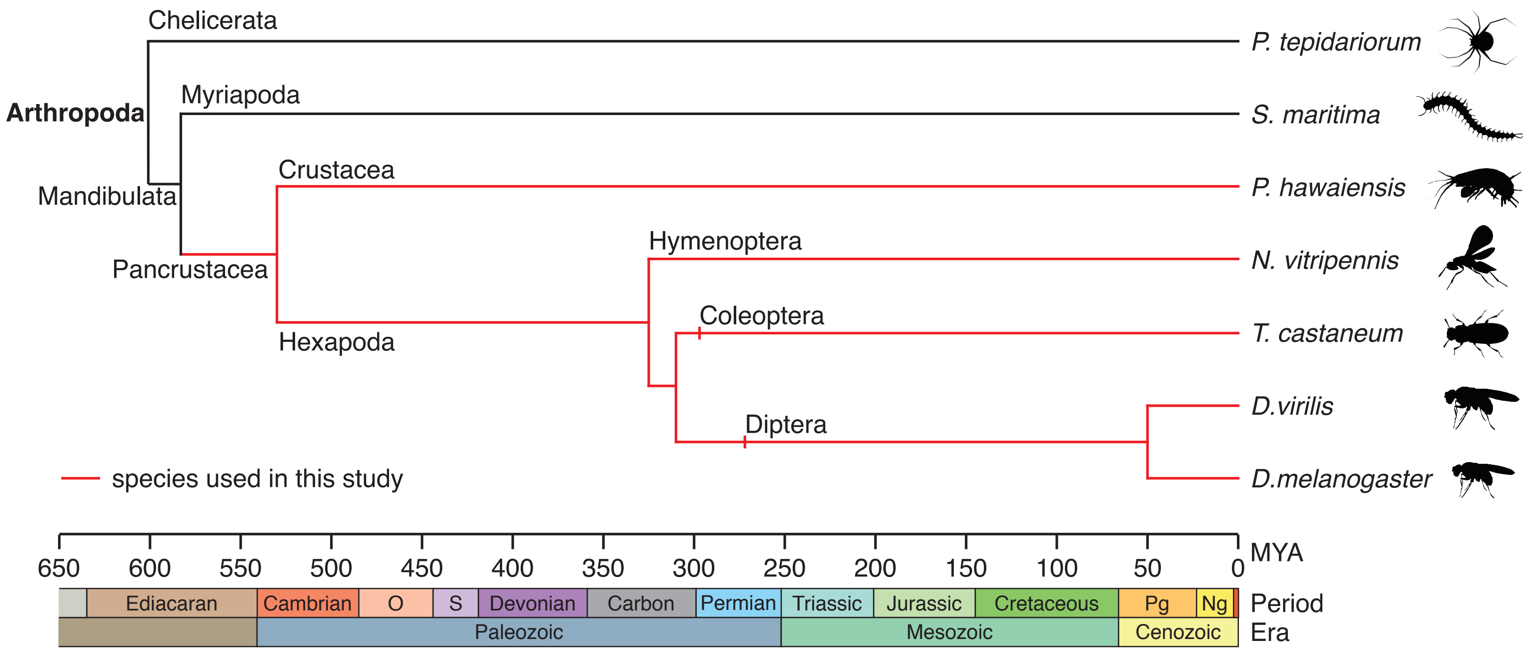

Supplementary Figure 1. Evolutionary divergence times of different arthropod model species. The species used in this study are highlighted in red, and belong to the clade pancrustacea, which emerged ~530 million years ago (MYA), and comprises all hexapods and crustaceans.

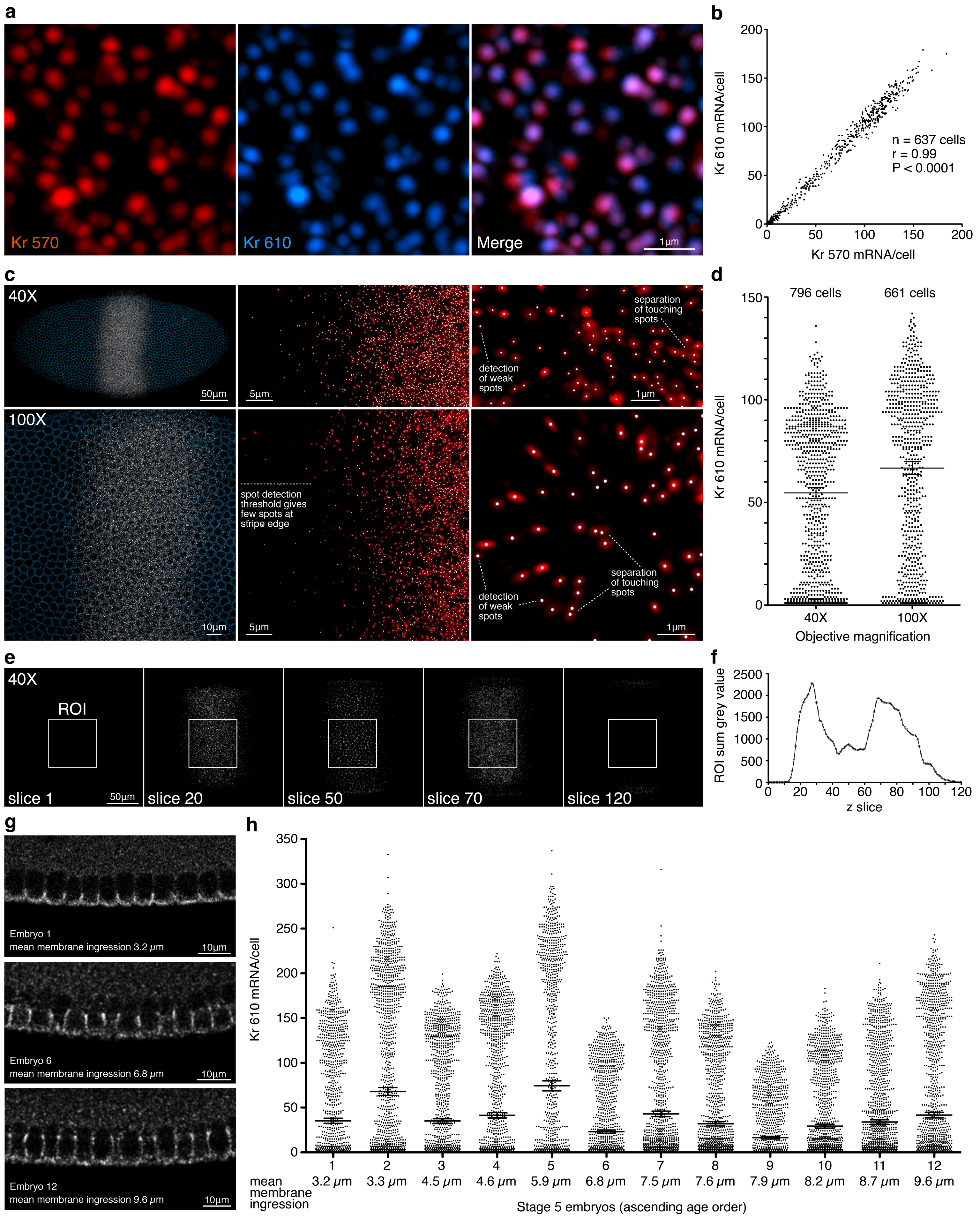

Supplementary Figure 2. Accuracy validations of mRNA quantification.

Supplementary Figure 2. Accuracy validations of mRNA quantification. a & b) Two colour detection efficiency test. **a)** smiFISH using two interleaved probe sets (each 41 probes) against *Kr* mRNA, labelled in Quasar 570 and CalFluor 610. Spots were imaged using 100X objective, through a 48 slice (total depth 9.6µm) z-stack. **b)** Spots in each channel were detected in Imaris, and assigned to the 637 segmented cells. Correlation in *Kr* mRNA/cell detected with each probe set was measured (two tailed Spearman ranked correlation coefficient $r = 0.99$, $P<0.0001$). **c & d)** Two magnification detection efficiency test. **c)** smiFISH of *Kr* mRNAs using CalFluor 610. The same stage 5 blastoderm embryo was imaged with 40X objective and then 100X objective, through the same 9.6µm z-depth (48 slices with z-step size of 200nm). Spot detection in Imaris using identical settings for both magnifications detected both strong and weak spots, successfully separated closely touching spots, with minimal false positives as indicated by the minimal spot detection towards the edge of the *Kr* stripe. **d)** *Kr* mRNA/cell at each magnification, non-zero cells only, each dot in the plot represents a cell, horizontal lines are the mean, error bars show the 95% confidence interval of the mean. There is a slight increase in detection efficiency at 100X compared with 40X (40X mean=54, max=136, 100X mean=66, max=142), showing a small degree of undercounting at 40X likely due to mRNA overcrowding. **e-h)** Biological variability in total *Kr* mRNA number. **e)** smiFISH of *Kr* mRNAs using CalFluor 610, imaged with 40X objective, beyond the basal limit of membrane ingression through a 120 slice (total depth 24µm) z-stack, to capture every *Kr* mRNA in the cytoplasmic depth. **f)** Slice by slice profile of sum greyscale intensity across a region of interest (ROI) through 120x 200nm z-slices. The trough between slices ~40-60 corresponds to the nucleus. **g)** Spectrin membrane staining at a cross sectional plane shows differing degrees of membrane ingression between stage 5 blastoderm embryos, used as a measure of embryo age. **h)** smiFISH of *Kr* mRNAs using CalFluor 610, in 12 blastoderm embryos imaged with 40X objective, z-step size of 200nm, through total z-depths ranging from 20µm to 24µm, set to extend from above the apical extent to below the basal extent of *Kr* mRNA spots. For cell segmentation, Spectrin membrane staining was extrapolated beyond the basal ingression limit, to the full stack depth. Identical Imaris spot detection settings were used across all embryos. The plot shows *Kr* mRNA/cell across the 12 blastoderm embryos arranged in ascending age order, as measured by the degree of membrane ingression. Each dot in the plot represents a cell, horizontal lines are the mean, error bars show the 95% confidence interval of the mean. Cell numbers: Embryo 1: 1526, 2: 1686, 3:1827, 4: 1923, 5: 1248, 6: 1797, 7: 1897, 8: 1834, 9: 1717, 10: 1641, 11: 1662, 12: 1830.
