# Supplementary material for: smiFISH and embryo segmentation for single-cell multi-gene RNA quantification in arthropods
Source: Commun Biol. 2021 Mar 19;4:352. doi: 10.1038/s42003-021-01803-0 (PMC7979837; doi:10.1038/s42003-021-01803-0)
Supplement: Supplementary file 3 — Description of Additional Supplementary Files [file 42003_2021_1803_MOESM3_ESM.pdf]

Description of Additional Supplementary Files

File name: Supplementary Data 1

Description: Gene-specific portions of all smiFISH probes used in this study. All sequences are written 5' to 3'. The common FLAP sequence – CCTCCTAAGTTTCGAGCTGGACTCAGTG – was added to the 5' end of each probe.

File name: Supplementary Data 2

Description: All probe-fluorophore combinations used in this study.
