# Supplementary material for: smiFISH and embryo segmentation for single-cell multi-gene RNA quantification in arthropods
Source: Commun Biol. 2021 Mar 19;4:352. doi: 10.1038/s42003-021-01803-0 (PMC7979837; doi:10.1038/s42003-021-01803-0)
Supplement: Supplementary file 6 — Reporting Summary [file 42003_2021_1803_MOESM6_ESM.pdf]

nature research

Corresponding author(s): Dr Tom Pettini

Last updated by author(s): 2021.01.18

Reporting Summary

Nature Research wishes to improve the reproducibility of the work that we publish. This form provides structure for consistency and transparency in reporting. For further information on Nature Research policies, see our Editorial Policies and the Editorial Policy Checklist.

Statistics

For all statistical analyses, confirm that the following items are present in the figure legend, table legend, main text, or Methods section.

n/a	Confirmed	
[ ]	[x]	The exact sample size (*n*) for each experimental group/condition, given as a discrete number and unit of measurement
[ ]	[x]	A statement on whether measurements were taken from distinct samples or whether the same sample was measured repeatedly
[ ]	[x]	The statistical test(s) used AND whether they are one- or two-sided<br>*Only common tests should be described solely by name; describe more complex techniques in the Methods section.*
[x]	[ ]	A description of all covariates tested
[x]	[ ]	A description of any assumptions or corrections, such as tests of normality and adjustment for multiple comparisons
[ ]	[x]	A full description of the statistical parameters including central tendency (e.g. means) or other basic estimates (e.g. regression coefficient) AND variation (e.g. standard deviation) or associated estimates of uncertainty (e.g. confidence intervals)
[x]	[ ]	For null hypothesis testing, the test statistic (e.g. *F*, *t*, *r*) with confidence intervals, effect sizes, degrees of freedom and *P* value noted<br>*Give P values as exact values whenever suitable.*
[x]	[ ]	For Bayesian analysis, information on the choice of priors and Markov chain Monte Carlo settings
[x]	[ ]	For hierarchical and complex designs, identification of the appropriate level for tests and full reporting of outcomes
[x]	[ ]	Estimates of effect sizes (e.g. Cohen's *d*, Pearson's *r*), indicating how they were calculated

Our web collection on statistics for biologists contains articles on many of the points above.

Software and code

Policy information about availability of computer code

Data collection	Leica LAS X v1.8.0.13370 software was used for image acquisition and spectral unmixing. Huygens Professional v18.04 software was used for image stabilization and deconvolution. Imaris v9.2 software was used to segment cells and quantify RNAs in images. FIJI v2.0.0-rc-49/1.51d software was used to project confocal image stacks.
Data analysis	Imaris v9.2 software was used to generate heat maps to display RNA number per cell. A custom R script was developed for neighbour finding using the package Simple Features for R (sf). Cell variability heat maps were generated in R using ggplot2.

For manuscripts utilizing custom algorithms or software that are central to the research but not yet described in published literature, software must be made available to editors and reviewers. We strongly encourage code deposition in a community repository (e.g. GitHub). See the Nature Research guidelines for submitting code & software for further information.

Data

Policy information about availability of data

All manuscripts must include a data availability statement. This statement should provide the following information, where applicable:

- Accession codes, unique identifiers, or web links for publicly available datasets
- A list of figures that have associated raw data
- A description of any restrictions on data availability

All smiFISH probe sequences are available in Supplementary Table 1. All smiFISH probe/fluorophore combinations are available in Supplementary Table 2. A high resolution image showing smiFISH staining for all eight Drosophila Hox genes is provided as Supplementary Image 1. These supplementary files, and all data underlying the graphs and heatmaps presented can be accessed at https://github.com/LliliansCalvo/smiFISH_Arthropods.

Field-specific reporting

Please select the one below that is the best fit for your research. If you are not sure, read the appropriate sections before making your selection.

- [x] Life sciences
- [ ] Behavioural & social sciences
- [ ] Ecological, evolutionary & environmental sciences

For a reference copy of the document with all sections, see nature.com/documents/nr-reporting-summary-flat.pdf

Life sciences study design

All studies must disclose on these points even when the disclosure is negative.

Sample size	Images and analysis presented in the main figures of this study are from single embryos of different species, or single tissues. This was considered a sufficient sample for the purposes of this methods paper, to demonstrate the methodology and analysis pipeline. In Supplementary Figure 2, biological variability was assessed between 12 different embryos, quantifying RNA in all segmented cells (~1000-2000 cells per embryo). All cell n numbers are stated in figure legends.
Data exclusions	As detailed in methods, some cells were eliminated during the whole embryo cell segmentation step. Specifically, edge cells, and any instances where the segmentation algorithm failed to split neighbouring cells, were omitted by filtering the set of detected cells for outliers based on cell volume, sphericity, and z-position.
Replication	smiFISH staining in each species was successfully performed multiple times, and on multiple embryos per experiment. Several smiFISH images were taken for each species and sample type, but a single example of each is presented in the main figures of the paper. Supplementary Figure 2 specifically assesses and confirms reproducibility, by quantifying the same gene in the the same embryo with two different probe sets, and at two different magnifications, and by analyzing 12 replicate embryos to assess biological variability.
Randomization	Randomization was not required for this study, because it demonstrates a methodology and analysis pipeline, and does not make biological comparisons requiring statistical tests.
Blinding	Blinding was not required for this study, because it demonstrates a methodology and analysis pipeline, and does not make biological comparisons requiring statistical tests.

Reporting for specific materials, systems and methods

We require information from authors about some types of materials, experimental systems and methods used in many studies. Here, indicate whether each material, system or method listed is relevant to your study. If you are not sure if a list item applies to your research, read the appropriate section before selecting a response.

Materials & experimental systems

n/a	Involved in the study	
☐	☒	Antibodies
☒	☐	Eukaryotic cell lines
☒	☐	Palaeontology and archaeology
☐	☒	Animals and other organisms
☒	☐	Human research participants
☒	☐	Clinical data
☒	☐	Dual use research of concern

Methods

n/a	Involved in the study	
☒	☐	ChIP-seq
☒	☐	Flow cytometry
☒	☐	MRI-based neuroimaging

Antibodies

Antibodies used	Mouse Monoclonal Anti Drosophila Alpha-Spectrin, Developmental Studies Hybridoma Bank, 3A9, Antibody Registry ID: AB_528473
Validation	Developmental Studies Hybridoma Bank reactivity statement: Positive tested species reactivity: Drosophila. Antibody reactivity in Drosophila was confirmed by successful immunofluorescence and imaging of cell membranes in Drosophila embryos, presented in this study.

Animals and other organisms

Policy information about studies involving animals; ARRIVE guidelines recommended for reporting animal research

Laboratory animals	Drosophila melanogaster, strain W1118, adult females, larvae and embryos<br>Drosophila virilis, wild-type embryos<br>Tribolium castaneum, wild-type embryos<br>Nasonia vitripennis, wild-type embryos

	Parhyale hawaiensis, wild-type embryos
Wild animals	The study did not involve wild animals.
Field-collected samples	The study did not involve samples collected from the field.
Ethics oversight	No ethical approval or guidance was required.

Note that full information on the approval of the study protocol must also be provided in the manuscript.
